# Immunologic Assessment of Tumors from a Race-matched Military Cohort Identifies Mast Cell Depletion as a Marker of Prostate Cancer Progression

**DOI:** 10.1158/2767-9764.CRC-22-0463

**Published:** 2023-08-01

**Authors:** Cara C. Schafer, Jiji Jiang, Sally Elsamanoudi, Darryl Nousome, Denise Y. Young, Yingjie Song, Isabell A. Sesterhenn, Gregory T. Chesnut, Shyh-Han Tan

**Affiliations:** 1 Center for Prostate Disease Research, Murtha Cancer Center Research Program, Department of Surgery, Uniformed Services University of the Health Sciences, Bethesda, Maryland.; 2 Henry M. Jackson Foundation for the Advancement of Military Medicine, Inc., Bethesda, Maryland.; 3 Frederick National Laboratory for Cancer Research, NCI, Frederick, Maryland.; 4 Joint Pathology Center, Silver Spring, Maryland.; 5 Urology Service, Walter Reed National Military Medical Center, Bethesda, Maryland.

## Abstract

Elucidating the cellular immune components underlying aggressive prostate cancer, especially among African American (AA) men who are disproportionately affected by this disease compared with Caucasian American (CA) men, will support more inclusive precision medicine treatment strategies. We aimed to evaluate which immune-related genes and cell types are differentially expressed in AA tumors and how immunobiology impacts prostate cancer progression.

We purified nucleic acid from tumor biopsies, obtained following radical prostatectomy, from 51 patients (AA = 26, CA = 25). Gene expression was measured using the NanoString platform from which we estimated immune cell abundances and assessed differences between groups based on clinicopathologic data. Product-limit estimates determined associations with biochemical recurrence (BCR)-free and metastasis-free survival.

*DVL2* and *KLRC2* were significantly upregulated in CA tumors and were also associated with worse disease progression. No significant differences in immune cell abundances by race were observed. Highly significant reductions in abundances of mast cells versus tumor-infiltrating lymphocytes (TIL) were found in men with high-grade pathologies and in men who later developed metastases. Low ratios of mast cells versus TILs were associated with worse BCR-free survival and metastasis-free survival.

Although estimated immune cell abundances were not different by race, we identified genes involved in metabolism and natural killer cell functions that were differentially expressed between AA and CA tumors. Among the entire cohort, depletion of mast cells within prostatectomy tumors was characteristic of advanced disease and susceptibility to disease progression.

**Significance:**

Our findings demonstrate that there are immune-related genes and pathways that differ by race. Impaired intratumoral cellular immune composition, especially for TIL-normalized mast cells, may be vital in predicting and contributing to prostate cancer disease progression.

## Introduction

There is an expanding interest to address health disparities in prostate cancer, notably among African American (AA) men who are disproportionately affected by increased risk, incidence, and mortality of this disease. Equitable access to care and other social determinants of health are known factors impacting prostate cancer disparity, yet even when these factors are accounted for, the exact biologic, epigenetic, and immunologic molecular mechanisms that may contribute to these differential incidences and outcomes for AA men are still not completely understood (1–6). This is due, in part, to limited availability of tumor specimens from AA men in these studies. Mindful of these considerations, we aimed to use specimens from men with equal access to health care in the Military Health System (MHS) to evaluate whether racial differences exist in the immunobiology of prostate cancer, especially among young AA men, and whether any immune cell profiles or specific immune-related genes are associated with disease progression. Emerging studies in this field have determined that AA men exhibit a unique immunobiologic tumor profile that may contribute to disease progression (2, 5), in addition to socioeconomic, environmental, and cultural factors. In recent years, efforts have been made to uncover any potential markers or changes in tumor biology distinguishing AA tumors from Caucasian American (CA) counterparts. Genomic studies have revealed marked differences in immune-oncologic and inflammatory pathways between AA and CA tumors (7–9), most notably that of cytokines (IL6, IL8, IFNγ) and metastasis-promoting genes (*AMFR*, *MMP9*; ref. 10). Weiner and colleagues (2021) discovered that immune content is augmented in AA prostate tumors, especially for natural killer (NK) cells and plasma cells, which was associated with improved outcomes (11). It has also been observed that CD24, a B-cell surface protein which is thought to functionally interact with mutant p53 to promote aggressive, metastatic prostate cancer, is expressed at significantly higher levels in AA men (12). Through the pathologic evaluation of T-cell density, Kaur and colleagues (2018) discerned there was no difference in the abundance of T cells based on racial ancestry (13). Yet, it has also been recognized that inflammatory infiltrates may be able to modulate the proinflammatory profile of prostate fibroblasts derived from AA men, more so than CA men (14). Similarly, the tumor-adjacent stroma, as opposed to tumor cells themselves, may be the source of differentially expressed genes regulating the extracellular matrix, epithelial-to-mesenchymal transition, and integrin signaling in AA tumor tissue (15). The prostate tumor microenvironment (TME) of AA men is also characterized by greater immunosuppression which could impair therapeutic effectiveness and lead to poorer outcomes among this demographic (16). All these factors combined are thought to contribute to aggressive prostate cancer in AA men.

Although immunotherapeutic effectiveness in prostate cancer has been less than satisfactory, it has been suggested that more efforts should be allocated toward understanding the prostate TME (17). Prostate cancer is considered to have a “cold” TME, lacking immunogenic stimuli and excessive tumor mutational burden, compared with many other primary cancer organ sites, complicating its treatment with precision medicine techniques. While this may be the case for most prostate cancers, there are exceptions, and subsets of localized prostate cancer with high densities of tumor-infiltrating immune cells could represent an opportunity for therapeutic intervention. Paradoxically, prostate cancer was the first organ site to obtain an FDA approval for use of an autologous immunotherapy, a therapeutic cancer vaccine called sipuleucel-T, for the treatment of castration-resistant prostate cancer (18). Trials have demonstrated that it extended overall survival but ultimately did not impair disease progression in the clinic (18). Sartor and colleagues (2020), however, found that AA men receiving sipuleucel-T had significantly improved overall survival compared with CA men when treated at lower baseline PSA levels (19), which may be due in part to observations that AA men have TMEs characterized by greater lymphocytic infiltration, proinflammatory cytokines, and lipid metabolism (7, 8, 10, 11). How these mechanisms influence immunotherapeutic response remains to be determined.

To address which immunobiologic factors may play a key role in prostate tumor progression, we conducted a pilot study to evaluate the immune relevance in cancer dissemination from a cohort with similar representation from AA and CA men. Utilizing specimens obtained from an equal-access MHS, we conducted gene expression studies to estimate the raw and relative abundances of immune cells and to measure the expression differences of immune-related genes as well as their impact on disease progression.

## Material and Methods

### Patient Specimens

We obtained written informed consent from Walter Reed National Military Medical Center (WRNMMC) patients under Institutional Review Board (IRB)-approved protocols for both the collection of their biospecimens in the Center for Prostate Disease Research (CPDR) Biobank (#393738) and of their clinical data in the Clinical Database (#GT90CM) for research. The work described herein was conducted in accordance with recognized ethical guidelines (e.g., Declaration of Helsinki, CIOMS, Belmont Report, U.S. Common Rule) and was approved as an exempt human study IRB protocol #DBS.2019.032 under the provision of 32 CFR 219.101(b) (4). Tumor specimens were obtained from patients undergoing radical prostatectomy (RP) at WRNMMC prior to treatment initiation. Fresh frozen, optimal cutting temperature–embedded tissue blocks were sectioned at 6-μm thickness. Representative hematoxylin and eosin–stained tissues were graded and marked by genitourinary pathologist (I.A. Sesterhenn). Tumor regions were isolated by macrodissection, and RNA was purified using the RNeasy Mini Kit (Qiagen). The Qubit RNA HS Assay Kit (Invitrogen) was used for RNA quantification.

### Gene Expression

Tumor RNA samples (1 ng input) were reverse transcribed to cDNA. Primer-specific regions were then linearly amplified through a multiplexed target enrichment approach using the Low RNA Input Kit (NanoString). We used both the Human chimeric antigen receptor (CAR)-T Panel (770 genes plus 10 internal reference genes) and the Human Immune Profiling panel (730 genes plus 40 internal reference genes), each containing probes for immune-related and housekeeping genes. Reaction products were hybridized to cartridges on the NanoString MAX/FLEX Prep Station, which were then scanned on the Digital Analyzer. Read counts were normalized to panel-specific reference genes that were selected using geNorm (20). Each panel was normalized independently and then merged results were prepared for downstream analyses in nSolver 4.0 with the Advanced Analysis plugin (version 2.0.134). To account for overlapping panel probes, nSolver uses a scaling method that takes the ratio of geometric means of all common probes between the two CodeSets. This ratio was then multiplied to the individual probe counts for both common and unique probes in the subsequent CodeSet. Counts for common probes were averaged in the merged data, while unique probe counts were adjusted by the ratio factor. The resulting values were used for downstream analyses.

### Immune Cell Abundance Estimation

RNA transcript deconvolution algorithms, based on previously published methods (21, 22), were applied using the nSolver Cell Type Profiling module to determine raw and relative immune cell abundance from known genes or gene sets. A score was generated on the basis of expression values, or an average log scale expression, of genes corresponding to a specific immune cell population. Raw cell type scores represented the log_2_ expression averages for each cell type's marker genes while relative cell type scores represented ratios of each cell type's raw score to either the total tumor-infiltrating lymphocytes (TIL) score (average of raw scores for B, T, CD45, macrophage, cytotoxic cells) or another cell subset's score [exhausted CD8, regulatory T cell (Treg), T cells]. These ratios, or relative scores, were then measured against each clinicopathologic variable of interest.

### Cohort Selection

An initial study cohort of self-reported AA (*n* = 30) and CA (*n* = 30) men with available frozen tissue at time of RP was selected by using propensity score matching for patients with the lowest differences in overall propensity scores derived from age at diagnosis, PSA at diagnosis, grade group (GG), and if they went on to develop metastasis. RNA was purified and used on the NanoString platform to evaluate gene expression. After excluding cases that had too few read counts or failed quality control (QC), a final cohort of 26 AA and 25 CA specimens was defined for downstream analysis. In this study, we use self-reported race, rather than genetic ancestry, to define patient race, mindful of the patient's selection and self-identification at time of consent.

### Statistical Tests

For comparison of the AA and CA cohorts, Fisher exact test was used for categorical variables and Wilcoxon two-sample test and median (IQR—interquartile range) statistics were used for continuous variables. To account for multiple comparisons testing of differential gene expression between AA and CA patient tumor specimens, the Benjamini–Yekutieli method was applied. This method yields moderately conservative estimates of FDR, assuming a biological connection between genes exists. Controlling and screening for FDR by resampling procedures using the Benjamini–Yekutieli method enables a more accurate comparison of multiple genes that may be inherently biologically and functionally related. GraphPad Prism 9 was used to evaluate relative cell type scores. We used the Mann–Whitney *U* test for two variables or groups as well as the Kruskal–Wallis test for trend and Dunn *post-hoc* test for multiple groups. To determine whether high or low gene expression was associated with BCR-free or metastasis-free survival, we used ROC analyses and the maximum value of the Youden index under the ROC curve to generate a cut-off point that equally optimizes the sensitivity and specificity of each gene or cell ratio score (23, 24). For comparison, we also computed log-rank *P* values derived from median cutoffs and HR *P* values derived from continuous variables. Kaplan–Meier curves were plotted using the *survminer* (v 0.4.9) R package (25), based on product-limit survival estimates and log-rank *P* values calculated by *SAS* version 9.4 (SAS Institute). All statistical tests were two sided and having a *P* value < 0.05 was considered significant. Overrepresentation analysis was evaluated using *ClusterProfiler* (v. 4.4.4; ref. 26) in the R programming environment.

### Data Availability

The datasets generated and analyzed in this study are not publicly available due to restrictions imposed by the current IRB protocol but can be made available from the corresponding author, upon approval of a separate IRB protocol and data sharing agreement allowing for their subsequent use.

## Results

### Patient Cohort Characteristics

Although the initial patient cohort was selected to be represented by both AA and CA patients that later progressed to develop BCR or metastasis, the final cohort, after excluding cases that failed QC, lacked any AA cases that developed metastasis. Prostate tumor specimens from 51 patients (Table 1) that passed QC filtering were evaluated by gene expression profiling of immune regulatory genes. Similar to patients with prostate cancer in the general population (27, 28) and within the MHS (29), the AA men in our current cohort received prostate cancer diagnoses at a much younger age compared with CA men, with a mean age of 56.8 and 62.2, respectively. Because the active-duty military population is substantially younger than the U.S. population (30), the CPDR biospecimen bank is enriched for a younger demographic from the outset. While the incidence of prostate cancer in AA men is also much higher in the military population compared with the general U.S. population, it has been speculated that this may be due to enhanced screening, detection, and access to care (30, 31). Importantly, a recent retrospective study of patients with low-risk prostate cancer in an equal-access health care setting supports the finding that neither AA nor CA race are predictors of BCR-free survival (32).

**TABLE 1:** Cohort summary

	AA (*N* = 26)	CA (*N* = 25)	*P*
Age group at diagnosis (years)			0.007
40s: 40–49	3 (11.5%)	3 (12.0%)	
50s: 50–59	14 (53.8%)	3 (12.0%)	
60s: 60–69	8 (30.8%)	15 (60.0%)	
70s: 70–79	1 (3.8%)	4 (16.0%)	
PSA group at diagnosis (ng/mL)			0.929
1: < 4	4 (15.4%)	4 (16.0%)	
2: 4–9	14 (53.8%)	12 (48.0%)	
3: > 9	8 (30.8%)	9 (36.0%)	
Gleason score			0.042
6	9 (34.6%)	5 (20.0%)	
7	12 (46.2%)	7 (28.0%)	
8	0 (0.0%)	5 (20.0%)	
9–10	5 (19.2%)	8 (32.0%)	
Grade group			0.020
Low: GG1 – GG2	21 (80.8%)	12 (48.0%)	
High: GG4 – GG5	5 (19.2%)	13 (52.0%)	
BCR			0.040
No	21 (80.8%)	13 (52.0%)	
Yes	5 (19.2%)	12 (48.0%)	
Metastasis			0.002
No	26 (100.0%)	17 (68.0%)	
Yes	0 (0.0%)	8 (32.0%)	
Diagnosis age (years)			0.006
Median (IQR)	56.3 (52.4–60.7)	64.0 (60.2–67.2)	
Diagnosis PSA (ng/mL)			0.993
Median (IQR)	6.6 (4.6–9.8)	6.3 (4.7–10.5)	

NOTE: Categorical variables were compared by Fisher exact test and continuous variables were evaluated by Wilcoxon two-sample test and median (IQR—interquartile range) statistics.

### Differential Gene Expression in AA and CA Tumors

**FIGURE 1:**
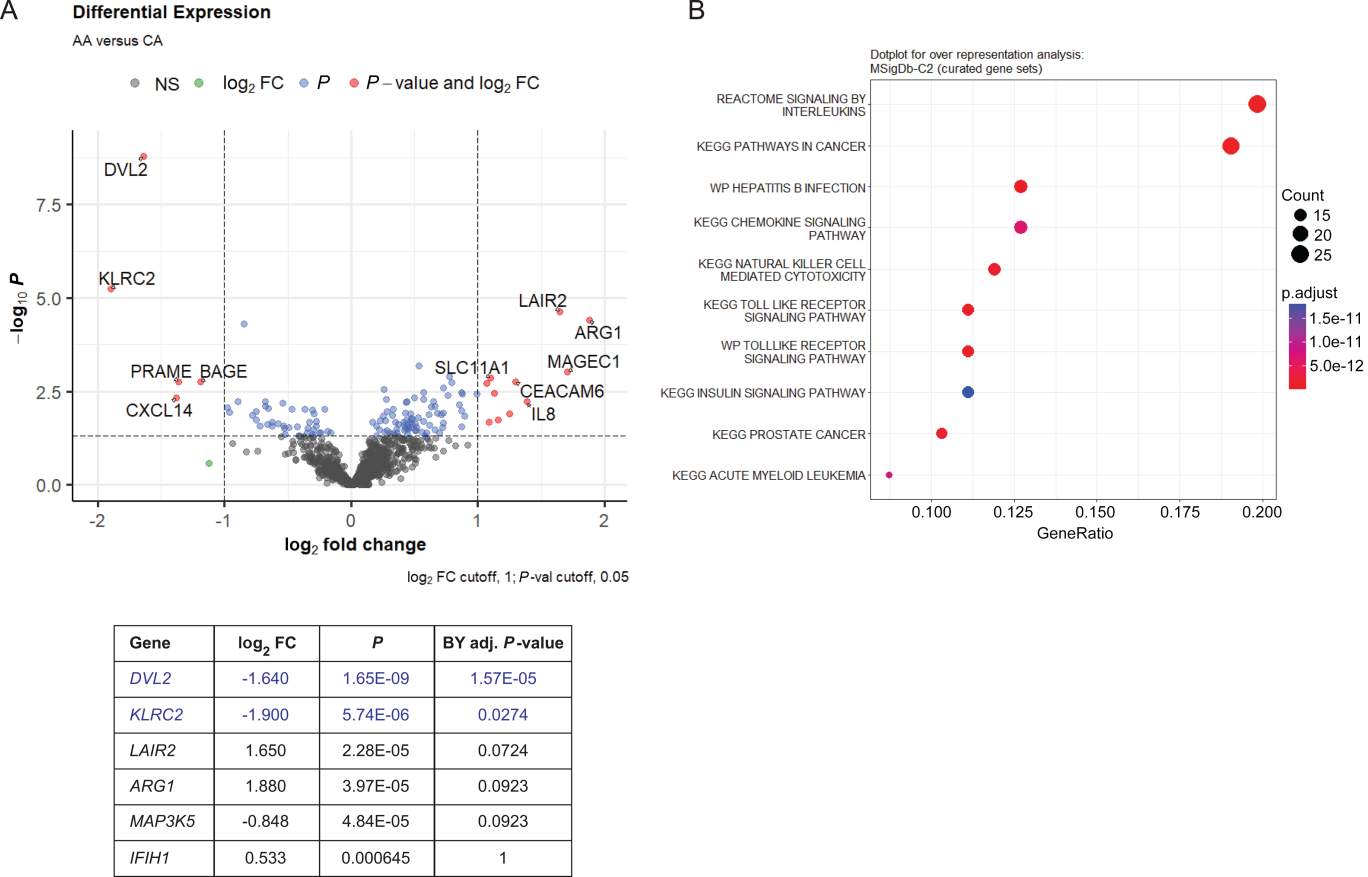
Differentially expressed immune-related genes in AA and CA tumors and pathway overrepresentation analysis. **A,** Volcano plot showing cutoffs for top differentially expressed genes in 26 AA tumors compared with 25 CA tumors as baseline. Red symbol genes in plot represent differentially expressed genes, with unadjusted *P* values < 0.05 and log_2_FC values either above 1 or below −1. FDR correction was applied, and only genes at or below Benjamini–Yekutieli adjusted *P* value of 1 are listed. Genes listed in blue text are significant following FDR correction at adjusted *P* value < 0.05. **B,** Dot plot highlighting top 10 relevant pathways detected by over representation analysis of 120 genes with differential expression *P* value < 0.05 using the MSigDb-C2 database.

We used the NanoString platform to evaluate gene expression differences between AA and CA tumors in promoting disease progression. Differential gene expression analysis of the combined data from the CAR-T and Immune Profiling panels identified 128 genes that were differentially expressed between AA and CA prostate tumors (*P*_unadjusted_ < 0.05; Supplementary Table S1). To control for type I errors, we used the Benjamini–Yekutieli method, setting the *P*_adjusted_ < 0.05 as statistically significant. We observed two genes, *DVL2* (dishevelled segment polarity protein 2) and *KLRC2* (killer cell lectin like receptor C2), that were significantly differentially expressed and downregulated in AA tumors, compared with CA tumors (Fig. 1A). *DVL2* is involved in metabolism via the mTOR, matrix metalloproteinase (MMP), and Notch/Wnt/β-catenin signaling pathways (33), while *KLRC2*, which encodes the natural killer cell receptor G2-C (NKG2C) receptor, has relevance in NK cell functions (34). When the MSigDb-C2 database was queried for overrepresentation assessment of the differentially expressed genes, we identified significant enrichment of genes involved in IL and chemokine signaling, NK cell–mediated cytotoxicity, and cancer signaling (Fig. 1B). Furthermore, when the differential expression results were mapped onto the Kyoto Encyclopedia of Genes and Genomes (KEGG) Pathways in Cancer network, *DVL2* and *IL8* showed the greatest AA versus CA fold changes (Supplementary Fig. S1).

### Evaluation of Top Differentially Expressed Genes and Their Association with Disease Progression

**FIGURE 2:**
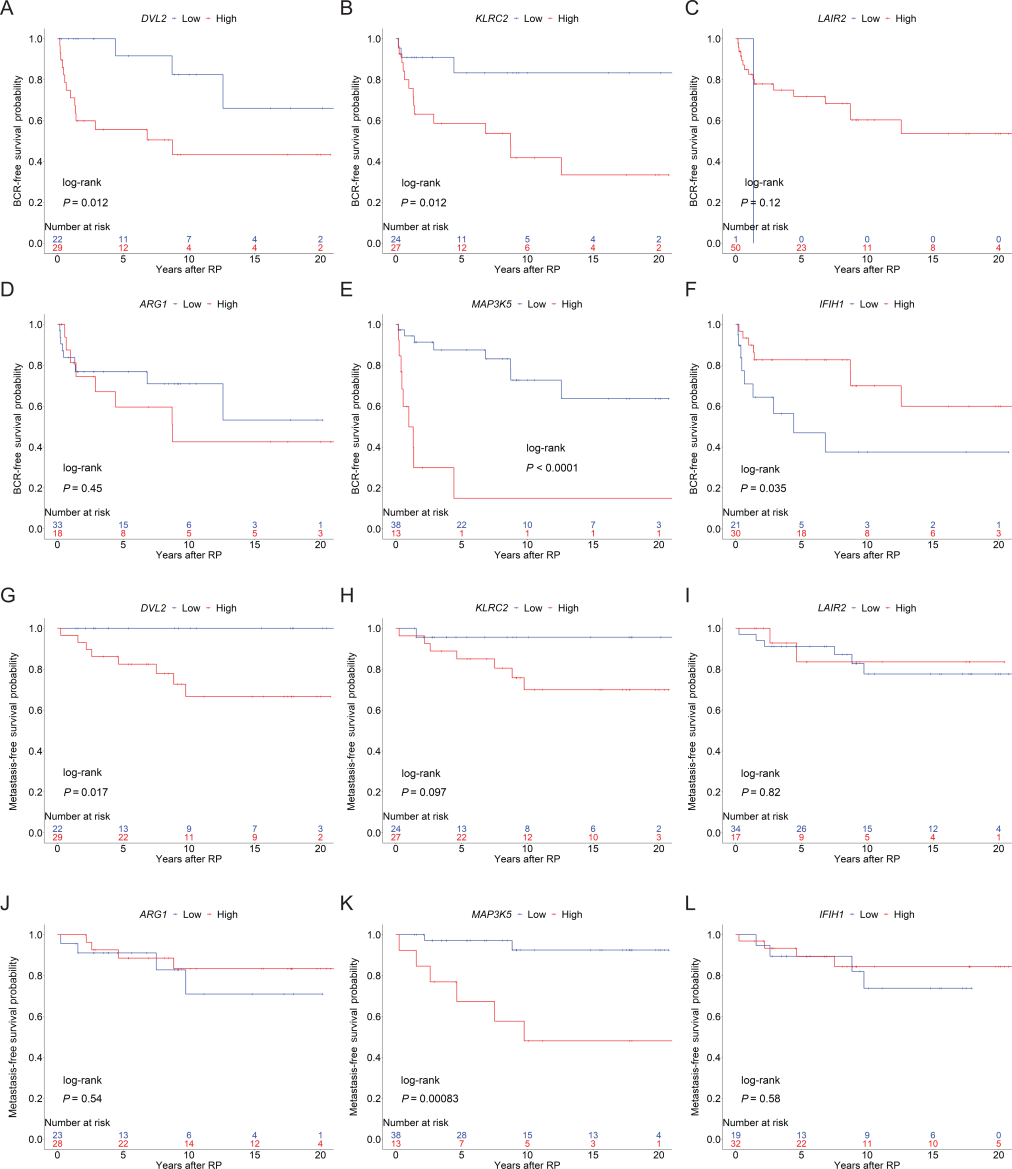
Top differentially expressed immune-related genes associated with BCR-free and metastasis-free survival. Kaplan–Meier product-limit survival curves are plotted for differentially expressed genes, *DVL2*, *KLRC2*, *LAIR2*, *ARG1*, *MAP3K5*, and *IFIH1*, and their association with BCR-free (**A–F**) and metastasis-free survival (**G–L**). Significant genes are indicated by log-rank *P* value < 0.05.

We then asked whether the top six differentially expressed genes in Fig. 1A, were associated with BCR-free (Fig. 2A–F) or metastasis-free (Fig. 2G–L) survival within the entire patient cohort. Shorter time to BCR was associated with high expression of *DVL2* (log-rank *P* = 0.012; Fig. 2A), *KLRC2* (log-rank *P* = 0.012; Fig. 2B), and *MAP3K5* (log-rank *P* < 0.0001; Fig. 2E). Meanwhile, low expression of *IFIH1* (interferon induced with helicase C domain 1) was associated with worse BCR-free survival (log-rank *P* = 0.035; Fig. 2F). Similarly, patients whose tumors had high expression of *DVL2* and *MAP3K5* were more likely to progress to metastatic disease (Fig. 2G and K; log-rank *P* = 0.017 and *P* = 0.000083, respectively). The association of the expression of these genes with BCR-free and metastasis-free survival were further evaluated as a continuous variable and as a categorical variable, classified by median cutoff (Supplementary Fig. S10; Supplementary Table S2). Expression of genes that were found to be significantly associated with BCR-free survival (*DVL2*, *KLRC2*, *MAP3K5,* and *IFIH1*) and metastasis-free survival (*DVL2* and *MAP3K5*), when analyzed as a continuous variable (HR *P* value < 0.05), were validated by, and in agreement with, the results of analysis as a categorical variable when patients were classified using the Youden index (log-rank *P* value < 0.05). On the basis of these observations, we anticipate that these genes and their related pathways may play key roles in prostate cancer disease progression. While low *DVL2* and *KLRC2* expression in AA tumors may be favorable, additional studies are warranted in a larger cohort with similar distributions of BCR and metastatic cases from CA and AA men.

### Immune Cell Profiles Based on Patient Clinicopathologic Features

**FIGURE 3:**
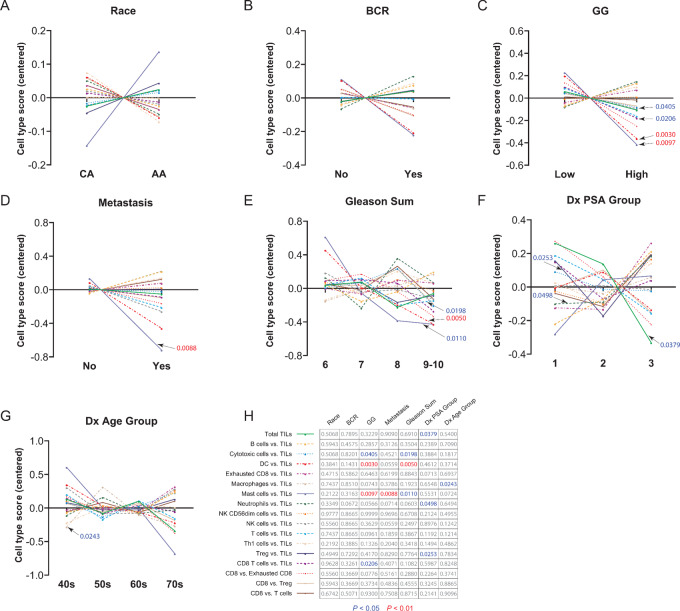
Immune cell profiling of prostate tumors based on clinicopathologic variables. Centered cell type scores are shown for race (**A**), BCR (**B**), GG (**C**), metastasis (**D**), Gleason sum (**E**), PSA at diagnosis (Dx PSA) group (**F**), and age (**G**) at diagnosis (Dx Age) group. **H,** Grid outlining cell type ratios for each clinicopathologic variable, based on Mann–Whitney *U* test (A–D) or Kruskal–Wallis test (E–G) at *P* values < 0.05 (blue), < 0.01 (red), and > 0.05 (gray).

The NanoString gene expression data were also used to evaluate immune cell type differences between AA and CA tumors and against available clinicopathologic features. We first examined the distribution of multiple immune cell types to TIL ratios, collectively, for each clinicopathologic variable. Figure 3 shows centered, relative cell type score comparisons that depict the ratio of the log_2_ scores for each cell type to total TILs. This ratio normalizes each sample to account for intersample variation in total tumor immune infiltrate that might be highly correlated with one cell type or another. Although we observed no significant difference in relative cell types for race or BCR (Fig. 3A and B; Supplementary Figures S2 and S3), mast cells versus TILs are slightly elevated in AA tumors, but this difference is not significant (*P* = 0.2122). Most strikingly, out of the remaining categories (Fig. 3C–G), both mast cells versus TILs and dendritic cell (DC) versus TILs scores were significantly lower in groups with higher GG (*P* = 0. 0097 and *P* = 0.0030, respectively; Fig. 3C) and DC versus TILs scores significantly lower in higher Gleason sums (*P* = 0.0050; Fig. 3E) groups compared with early-stage prostate tumor pathologies. Mast cells versus TILs in metastatic cases, consisting of only CA cases, were also significantly diminished (*P* = 0.0088; Fig. 3D), indicating a role in cancer progression. The grid in Fig. 3H lists *P* values for all group comparisons.

**FIGURE 4:**
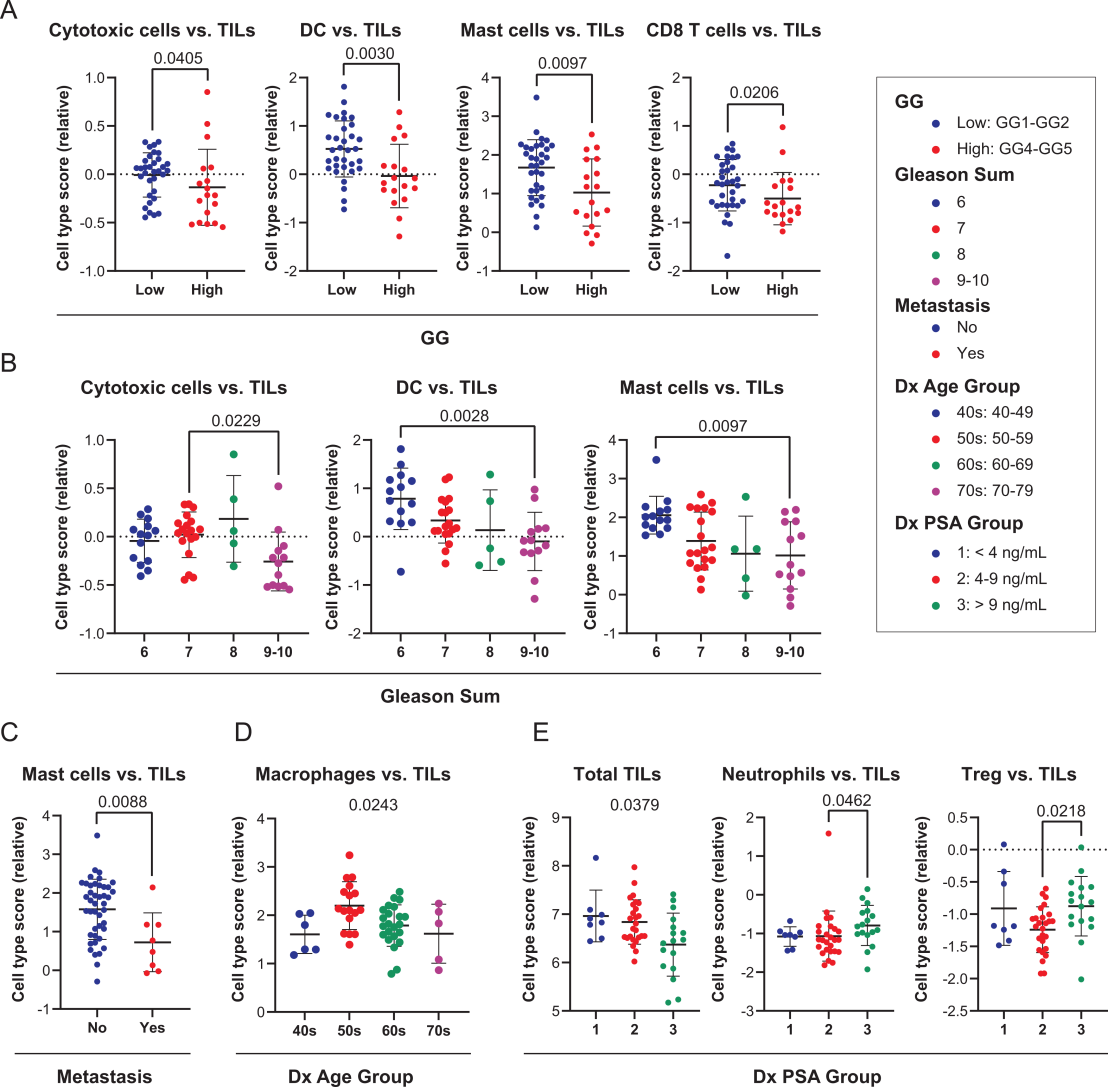
Significant relative cell type scores for clinicopathologic variables. Relative cell type scores that demonstrated significance for GG (**A**), Gleason sum (**B**), metastasis (**C**), age at diagnosis group (**D**), and PSA (**E**) at diagnosis group are shown. Mann–Whitney *U* test (A, C) or Kruskal–Wallis test followed by Dunn multiple comparisons *post-hoc* test (B, D, E) were used to determine significance, as indicated by listed *P* values below 0.05. Each dot represents a patient score, and error bars correspond to mean ± SD.

We further highlight individual immune cell populations that demonstrated significant differences in immune cell to TILs ratios across all clinicopathologic variable categories, shown as column scatter plots in Fig. 4. Relative scores for cytotoxic cells, DC, mast cells, and CD8 T cells versus TILs were all significantly lower in the high GG group (Fig. 4A; *P* = 0.0405, *P* = 0.0097, and *P* = 0.0206, respectively), suggesting that their presence is depleted in advanced stage tumors. Similarly, cytotoxic cells, DC, and mast cells versus TILs scores were significantly lower in relative abundance within Gleason sum 9–10 patient tumors (Fig. 4B; Dunn *post-hoc* test *P* = 0.0229, *P* = 0.0028, and *P* = 0.0097, respectively). Mast cells versus TILs were significantly less abundant in tumors that later went on to develop metastatic disease (Fig. 4C; *P* = 0.0088). Although macrophages versus TILs trended lower with increasing patient Dx Age (Kruskal–Wallis test *P* = 0.0243), there was no significant difference in relative scores between individual Dx Age Groups (Fig. 4D). In Fig. 4E, the abundance of total TILs diminished with increasing Dx PSA (Kruskal–Wallis test *P* = 0.0379), yet neutrophil and Treg versus TIL scores were significantly elevated in Dx PSA Group 3 versus Dx PSA Group 2 (Dunn *post-hoc* test *P* = 0.0460 and *P* = 0.0218, respectively). Additional graphs of clinicopathologic features for remaining cell type ratios that were not statistically significant can be found in Supplementary Figs. S2–S8.

### Cell Types Associated with BCR-free and Metastasis-free Survival

Finally, we addressed whether high or low relative cell type scores were associated with BCR-free and metastasis-free survival as a continuous variable and as a categorical variable, grouped by median, and by Youden index cutoffs (Supplementary Table S3). We observed that total TILs were not significantly associated with shorter time to BCR (Supplementary Fig. S9A and S9B) or metastasis (Supplementary Fig. S9C and S9D), both when classified by sample median or by Youden index. These results indicate that evaluations of the association between ratios of individual cell types to total TILs and BCR or metastasis will not be confounded by any differences in total TILs.

**FIGURE 5:**
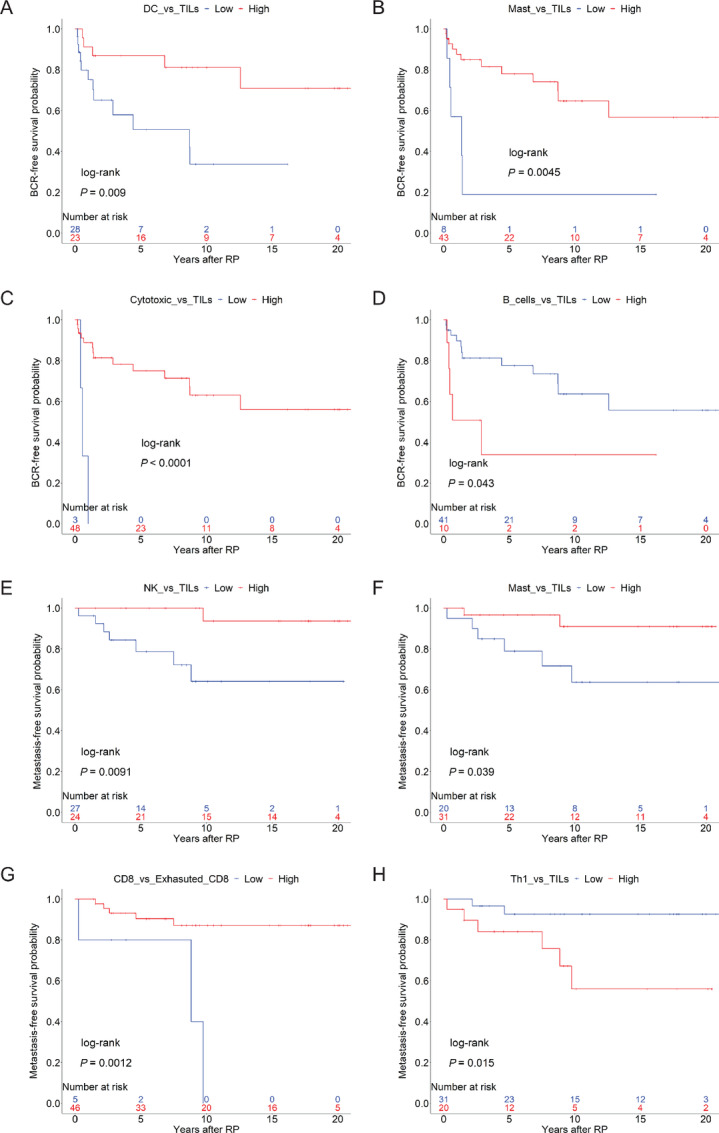
BCR-free and metastasis-free survival probabilities for cell type ratios with clinical significance. Youden index is used to stratify high versus low abundances. BCR-free survival probabilities are plotted for high and low relative cell type scores for DC (**A**) versus TILs, mast cells versus TILs (**B**), cytotoxic cells versus TILs (**C**), and B cells versus TILs (**D**) based on log-rank *P* < 0.05. Metastasis-free survival probabilities are shown for high and low relative cell type scores for NK cells versus TILs (**E**), mast cells versus TILs (**F**), CD8 versus exhausted CD8 (**G**), and Th1 versus TILs (**H**) based on log-rank *P* < 0.05.

Shorter time to BCR, however, was associated with low relative abundance scores for DC versus TILs (log-rank *P* = 0.009; Fig. 5A), mast cells versus TILs (log-rank *P* = 0.0045; Fig. 5B), and cytotoxic cells versus TILs (log-rank *P* < 0.00001; Fig. 5C), and high relative abundance scores for B cells versus TILs (log-rank *P* = 0.043; Fig. 5D). Significant associations between shorter time to BCR and low DC versus TILs, as well as low mast cells versus TILs, were also observed when the analyses were performed using the relative abundance cell type scores as a continuous variable (*P* = 0.00676 and *P* = 0.02374, respectively) and as a categorical variable dichotomized by sample median (*P* = 0.02776 and *P* = 0.03926, respectively; Supplementary Table S3; Supplementary Fig. S11A–S11D).

Low abundance scores for NK versus TILs and mast cells versus TILs portended worse metastasis-free survival (Fig. 5E and F; log-rank *P* = 0.0091 and *P* = 0.039, respectively). Significant associations between shorter time to metastasis and low mast cells versus TILs, as well as low NK versus TILs, were also observed when the analyses were performed using the relative abundance cell type scores as a categorical variable dichotomized by sample median (*P* = 0.01606 and *P* = 0.03424, respectively; Supplementary Table S3; Supplementary Fig. S11E–S11H). When analyzed as a continuous variable, low mast cells versus TILs, and low DC versus TILs were found to be associated with shorter time to metastasis (*P* = 0.01119 and *P* = 0.01603, respectively; Supplementary Table S3).

Interestingly, low ratios of CD8 to exhausted CD8 T cells were also associated with a greater likelihood of developing metastasis (Fig. 5G; log-rank *P* = 0.00124) while tumors with low Th1 versus TILs scores had better metastasis-free survival (Fig. 5H; log-rank *P* = 0.0153). Notably, low relative abundance scores for mast cells versus TILs were associated with both worse BCR-free and metastasis-free survival (Fig. 5B and F), which suggests that low ratios of mast cells to TILs may be highly predictive of worsening disease. This was also true for mast cells versus TILs when a median cutoff was used (Supplementary Fig. S11A–S11H).

A feature of our cohort is that more AA men are younger and have lower Gleason sum and GG tumors, compared with CA men. To resolve whether race may be driving the observed significant difference in mast cells versus TIL scores with increasing Gleason sum and GG, we evaluated each clinical grouping (e.g., 60s for Dx Age, low for GG). As shown in Supplementary Fig. S12A–S12C, we see that mast cells versus TIL scores are not significantly different between each grouping for Gleason sum, in high versus low GG, or between each grouping for Dx Age, indicating that AA race is not likely to impact the observed outcomes.

## Discussion

While socioeconomic factors are major contributors to health disparities in cancer, it is evident that equal access to care mitigates racial discrepancies in prostate cancer mortality, and this results in comparable prostate cancer survival rates between AA and CA men (29, 35). Institutions with equal access to care are uniquely positioned to evaluate whether health disparities are influenced by intrinsic factors, such as biological and genetic elements, which might otherwise be obscured by preexisting social determinants of health. Determining the baseline immune profiles, integrated with available clinicopathologic data, will help us to better understand the prostate cancer tumor immune microenvironment and guide future treatment approaches. Recent advances in immunotherapy and precision medicine in cancer therapy strategies make this a promising pursuit.

On the basis of our gene expression studies, and using a stringent adjusted *P*-value cutoff, we identified two genes, *DVL2* and *KLRC2*, that were significantly downregulated in AA tumors. It is important to note that bulk gene expression analyses cannot definitively discern the cell type that is expressing a gene of interest, although existing data on known gene functions and correlative studies can presume their role in one cell type over another. The roles of *DVL2* in mTOR and Wnt/β-catenin signaling and *KLRC2* in NK cell functions suggest that biological mechanisms associated with metabolism and antitumor NK-cell pathways are dampened in AA tumors. *DVL2* has been shown to enhance the metastatic potential of prostate cancer by upregulating expression of Wnt-3a, AR, and MMP (33). This is also consistent with the findings of Rayford and colleagues (2021) that CA men had higher levels of Wnt/β-catenin signaling activation (36). Tumor-intrinsic Wnt/β-catenin activation has been shown to be associated with impaired chemokine production and inhibition of DC recruitment, leading to immune evasion and resistance to immune checkpoint blockade across a range of cancers (37, 38). Within our cohort, high expression of *KLRC2* was associated with worse BCR-free survival, while high *DVL2* was associated with both worse BCR-free and metastasis-free survival. Merino and colleagues (2019) showed that chronic stimulation of NK cells using anti-NKG2C agonistic antibodies have less effector function and express elevated immune checkpoint inhibitory molecules (34). This seems to suggest that *KLRC2*-mediated NKG2C activation and the subsequent upregulation of immune checkpoint molecules on NK cells may aid in circumventing immune surveillance. Interestingly, Goncalves and colleagues (2016) have shown that a higher frequency of homozygous *KLRC2* deletion is found in West African versus East African populations (39) and represents a race-associated mechanism that could be explored further. Downregulation of *DVL2* and *KLRC2* in AA tumors might suggest more efficient regulation of metastasis-promoting pathways as well as NK-cell exhaustion. *MAP3K5* is known to be involved in tumorigenesis and inflammatory processes and its expression is elevated in high-risk prostate cancer (40), consistent with our observations. Similarly, *IFIH1*, which encodes the MDA5 viral pathogen sensor implicated in IFN and innate immune responses, has been highlighted by others as a gene whose variants can affect cancer IFN signaling and, potentially, response to immunotherapy (41). Overrepresentation analysis of the differentially expressed genes identified significant involvement of IL, chemokine, and Toll-like receptor signaling as well as NK cell–mediated cytotoxicity. These differences in immune response and chemokine signaling have been identified in prior studies (7, 9, 10). Additional pathway mapping using the KEGG database showed that *DVL2* and its associated genes were downregulated in AA tumors. We also detected higher IL8 expression in AA tumors, which has been shown to be associated with prostate cancer aggressiveness (42) and is known to be produced by angiogenic and tumor-promoting mast cells (43). It would be worthwhile to explore the relevance of these pathways impacting overall survival and disease progression within the general population to determine whether they are biological contributors to the etiology of prostate cancer.

For the evaluation of immune cell profiles, we used the relative cell abundances (ratio of each cell type to total TILs or to other individual cell types) plotted for each available clinicopathologic variable. Although we anticipated a significant difference in suppressive and lymphocytic immune cell types between AA and CA men, based on earlier reports in AA tumors, this was not seen in our cohort. This finding could be attributed to a difference in equal access to care or to a difference in Dx Age, because the AA men in this study cohort, and overall, within the CPDR database, are diagnosed at a much younger age compared with CA men. This does not discount the presence of existing germline or innate differences that may contribute to development of earlier and aggressive disease.

Mast cells versus TIL scores were significantly lower for GG, Gleason sum, and metastasis variables, which suggests they may be sensitive to prostate cancer initiation and dissemination. In addition, DC, cytotoxic cells, and CD8 T cells versus TIL scores trended significantly lower with increasing GG and Gleason sum, indicating a pathobiological role for these cells in tumor immunity. The macrophages versus TILs were the only ratios associated with Dx Age Group, suggesting that macrophages may be important in the timing of the onset of disease. Finally, the total TILs abundance decreased with increasing Dx PSA, but neutrophil and Treg versus TIL scores were elevated in Dx PSA Group 3 versus Dx PSA Group 2. These two cell types may be enriched in high PSA patients and could represent cellular targets for monitoring or prioritizing treatment in patients presenting with high PSA values (>9 ng/mL) at RP.

Overall cohort assessment revealed that relative abundances of mast cells were significantly lower in cases that developed metastasis, consisting of all CA cases, and in cases that had a higher GG as well as Gleason sum at diagnosis. Previous studies have identified both proinflammatory and anti-inflammatory roles of mast cells in prostate cancer progression which could be dependent on their location in the TME. Elevated intratumoral mast cell density has been found to be associated with favorable prognosis in prostate cancer (44, 45) and Nonomura and colleagues (2007) found that aggregation of mast cells around tumor foci from needle biopsies are prognostic of poorer outcome in prostate cancer (46). More recent studies by Hempel and colleagues (2020) have shown that high counts of mast cells in the extratumoral compartment were associated with greater risk of BCR and metastasis but that intratumoral mast cell counts had no association (47). Notably, they did find a significant reduction in both intratumoral and extratumoral mast cells in AA men compared with CA men, but this association disappeared when stratified by other clinical variables (47). Subsequent studies revealed an important role of extratumoral tryptase-containing mast cells in adverse disease outcomes following RP (48). In alignment with these reports, we observe a reduction in mast cell versus TIL abundance with high GG, high Gleason sum, and in patient tumors that later developed metastatic disease. Although we did not detect a significant reduction in mast cells versus TIL abundance with preoperative PSA, as found by Fleischmann and colleagues (2009) in microarray studies (45), we did observe that low mast cells versus TIL abundance was associated with worse BCR-free and metastasis-free survival probability. Thus, it is reasonable to conclude that the absence of these cells, as a ratio to the total immune infiltrate, in the TME may be contributing to tumoral immune escape leading to disease progression.

Like mast cells, DCs versus TILs and cytotoxic cells versus TILs scores were also significantly lower in patients with high GG and Gleason sum. As DCs are critical for antigen presentation to T cells to elicit an antitumor immune response, suppression of these cells in the TME with respect to the total tumor-infiltrating lymphocytes may lead to tolerance or immune escape allowing the tumor to propagate (49). Patients with a lower proportion of cytotoxic cells, DCs, mast cells, or NK cells in tumors at RP have much worse BCR-free or metastasis-free survival outcomes. These observations are consistent with our initial finding of low cell type scores for cytotoxic cells versus TILs, DC versus TILs, and mast cells versus TILs in high GG and Gleason sum cases and suggest a strong relationship between the loss of these cells in the TME and disease progression. Likewise, others have found that the presence of NK cells and their tumor-lytic effects have favorable prognoses in patients with metastatic prostate cancer (50). Intriguingly, B cell and Th1 versus TIL scores were not significantly different based on pathology or clinical features at diagnosis, but high scores were associated with shorter time to BCR and metastasis, respectively. There is some evidence to support this as B cells have been reported to be intratumorally enriched in high-risk prostate cancer (51), although others have described the presence of plasma cells in tumors with improved outcomes, especially among AA men (11). Although Th1 cells are critical in sustaining an antitumor immune response via IFNγ signaling (52), we observe that high tumor Th1 versus TIL scores progressed to metastatic disease. While this study does not assess subtypes within a particular cell type, this may be an important consideration in justifying these discrepancies. Patients whose tumors had low CD8 to exhausted CD8 scores, or greater abundance of exhausted CD8 T cells, were also more likely to progress to metastatic disease which supports CD8 T-cell anergy and exhaustion as a plausible tolerance mechanism in tumoral immune escape.

In addition to evaluating our data using the Youden index and median cutoffs, we also calculated the HR *P* values from each dataset as a continuous variable. There was consistent overlap in survival outcomes for *DVL2*, *KLRC2*, *MAP3K5*, and *IFIH1* as well as for mast cells versus TILs. By using the Youden index to dichotomize patients in analyses of the data as a categorical variable, we identified significant associations with BCR and metastasis that concur with those identified by analysis of the data as a continuous variable or as a categorical variable grouped by sample median. It will be important to further investigate how the genes and immune cell subsets may contribute to both the onset and the progression of prostate cancer in future studies using larger patient cohorts. Others have highlighted the importance of the location and function of mast cells in the tumor and surrounding prostate compartments and how they elicit opposing effects depending on their spatial distribution (48, 53). Forthcoming studies should aim to focus on the spatial distribution and functional characterization of mast cells, as well as B cells, Th1 cells, NK cells, and DCs in the prostate TME.

The primary limitation of our study is that our final, evaluable cohort is unbalanced. Because of this, we cannot make any race-dependent claims on outcome. The strengths of our study include a comprehensive tumor profiling of immune-related genes and immune cell types within a patient cohort that has nearly equivalent distributions of AA and CA men. Only a small amount of input RNA, as little as 1 ng, was needed from biopsy tissue to generate these results, which provides a proof-of-concept for assessment of patient immune infiltration from minimal tumor biopsy tissue. When used in conjunction with targeted MRI-PET–guided biopsy methods to visualize the prostate tumor, this may be a unique companion method to evaluate immune profiles in clinical studies with limited patient specimens.

In conclusion, we observe no significant difference in immune cell composition with respect to self-reported race, although metabolism, Wnt/β-catenin signaling, and NK-cell activities are differentially regulated in AA versus CA tumors, pointing to possible cellular functional differences. Assessment of immune profiles of the entire cohort reveals a significant role for mast cell depletion in prostate cancer disease pathology. The difference in ratios of mast cells to total TILs in BCR-free and metastasis-free survival outcomes also suggests that the interaction between mast cells and TILs in the TME may be critical for immunosurveillance and cell-to-cell interactions needed for antigen presentation and T-cell activation. Furthermore, the intratumoral loss of these cells in high-grade prostate cancer may serve as a prognostic marker, and potentially as a therapeutic target in rescuing these cells, in patients with late-stage prostate cancer.

## Supplementary Material

Supplementary Figure S1 Supplementary Figure S1 shows differentially expressed gene pathways in AA tumors.

Supplementary Figure S2 Supplementary Figure S2 shows non-significant relative cell type scores by race.

Supplementary Figure S3 Supplementary Figure S3 shows non-significant relative cell type scores by BCR status.

Supplementary Figure S4 Supplementary Figure S4 shows non-significant relative cell type scores by GG.

Supplementary Figure S5 Supplementary Figure S5 shows non-significant relative cell type scores by metastasis status.

Supplementary Figure S6 Supplementary Figure S6 shows non-significant relative cell type scores by Gleason Sum.

Supplementary Figure S7 Supplementary Figure S7 shows non-significant relative cell type scores by Dx PSA Group.

Supplementary Figure S8 Supplementary Figure S8 shows non-significant relative cell type scores by Dx Age Group.

Supplementary Figure S9 Supplementary Figure S9 shows BCR-free and Metastasis-free survival for Total TILs scores dichotomized by median as well as Youden index cutoffs.

Supplementary Figure S10 Supplementary Figure S10 shows BCR-free and Metastasis-free survival for top differentially expressed genes in AA tumors dichotomized by median cutoffs.

Supplementary Figure S11 Supplementary Figure S11 shows BCR-free and Metastasis-free survival for cell types dichotomized by median cutoffs.

Supplementary Figure S12 Supplementary Figure S12 shows Mast vs TILs scores plotted by race for Gleason Sum, GG, and Dx Age.

Supplementary Table S1 Supplementary Table S1 shows the full list of differentially expressed genes in AA tumors along with log2 fold changes, p-values, and adjusted p-values.

Supplementary Table S2 Supplementary Table S2 shows gene expression evaluated as a continuous variable and by median and Youden index cutoffs for BCR-free and metastasis-free survival.

Supplementary Table S3 Supplementary Table S3 shows Total TILs and individual cell type vs TILs scores evaluated as a continuous variable and by median and Youden index cutoffs for BCR-free and metastasis-free survival.
