# Supplementary material for: Immunologic Assessment of Tumors from a Race-matched Military Cohort Identifies Mast Cell Depletion as a Marker of Prostate Cancer Progression
Source: Cancer Res Commun. 2023 Aug 1;3(8):1423–34. doi: 10.1158/2767-9764.CRC-22-0463 (PMC10392708; doi:10.1158/2767-9764.CRC-22-0463)
Supplement: Supplementary Figure S1 — shows differentially expressed gene pathways in AA tumors. [file crc-22-0463-s01.pdf]

Supplementary Figure S1

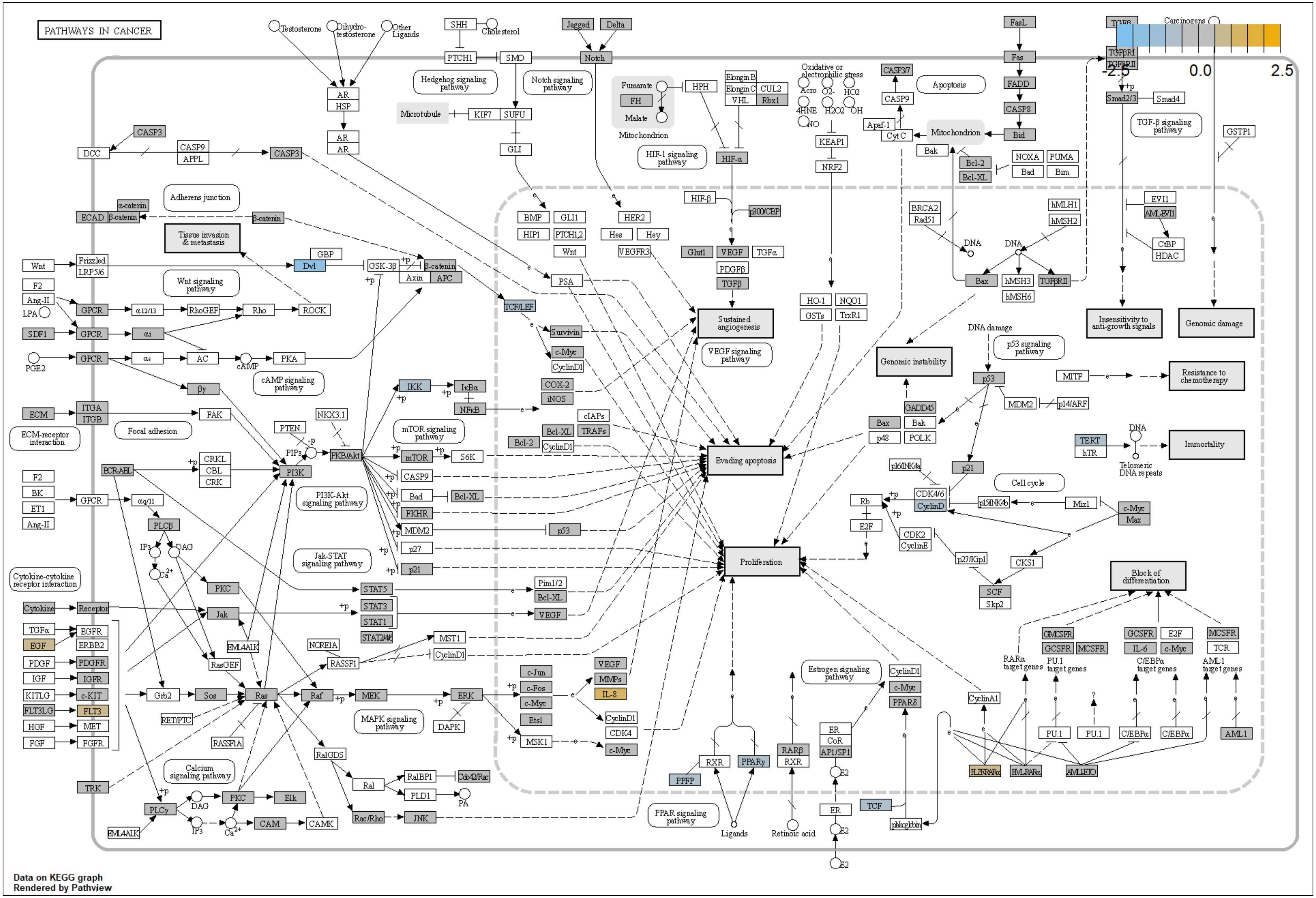

Supplementary Figure S1. KEGG Pathways in Cancer diagram highlighting relevant fold change gene expression differences in AA tumors.
