# Supplementary material for: Immunologic Assessment of Tumors from a Race-matched Military Cohort Identifies Mast Cell Depletion as a Marker of Prostate Cancer Progression
Source: Cancer Res Commun. 2023 Aug 1;3(8):1423–34. doi: 10.1158/2767-9764.CRC-22-0463 (PMC10392708; doi:10.1158/2767-9764.CRC-22-0463)
Supplement: Supplementary Figure S2 — shows non-significant relative cell type scores by race. [file crc-22-0463-s02.pdf]

Supplementary Figure S2

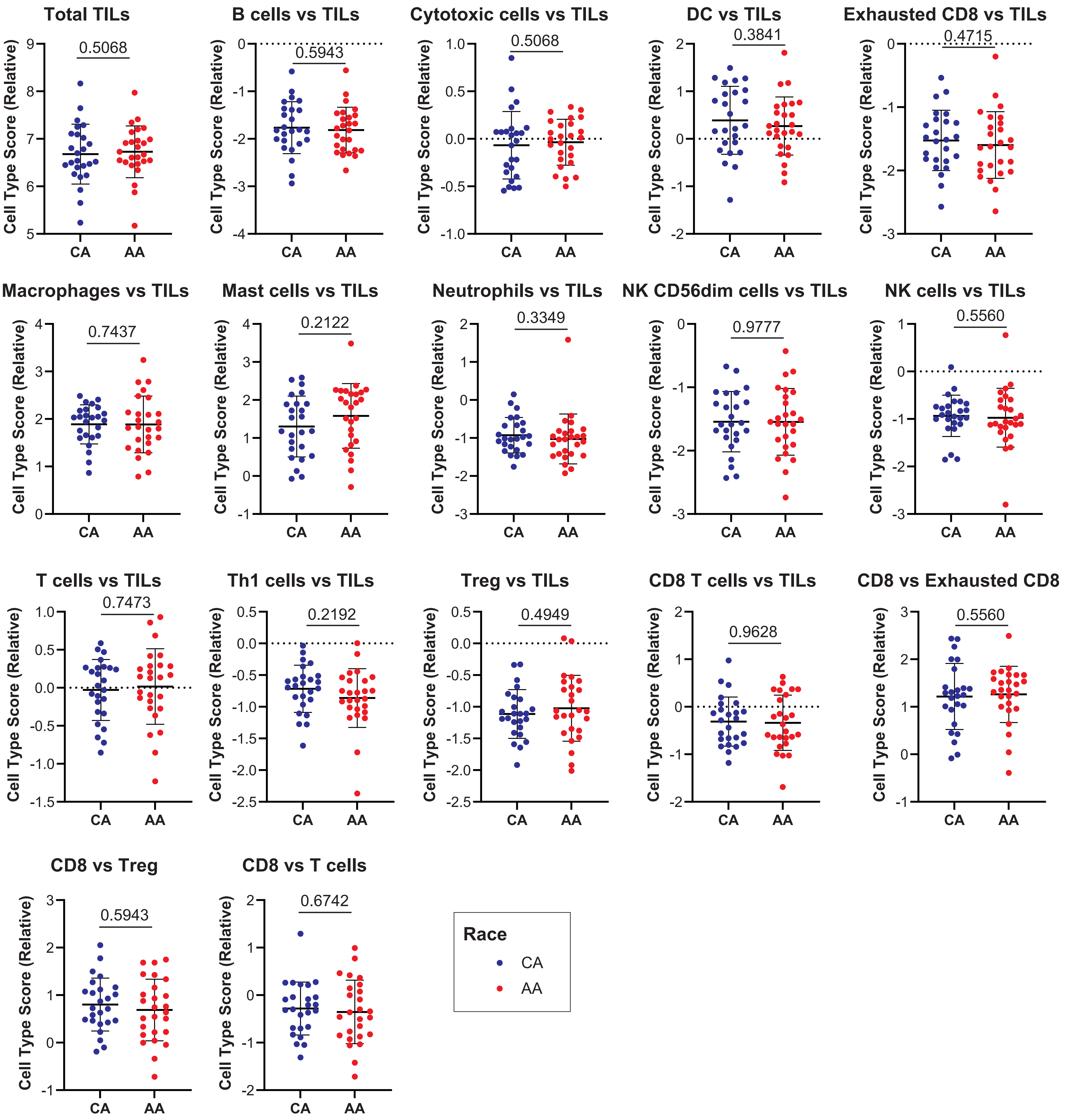

Supplementary Figure S2. Additional relative cell type contrasts compared by race. Individual relative cell type scores are plotted for AA and CA tumors and are evaluated by Mann-Whitney U test. Each dot represents a patient score, and error bars correspond to mean ± SD.
