# Supplementary material for: Immunologic Assessment of Tumors from a Race-matched Military Cohort Identifies Mast Cell Depletion as a Marker of Prostate Cancer Progression
Source: Cancer Res Commun. 2023 Aug 1;3(8):1423–34. doi: 10.1158/2767-9764.CRC-22-0463 (PMC10392708; doi:10.1158/2767-9764.CRC-22-0463)
Supplement: Supplementary Figure S4 — shows non-significant relative cell type scores by GG. [file crc-22-0463-s04.pdf]

Supplementary Figure S4

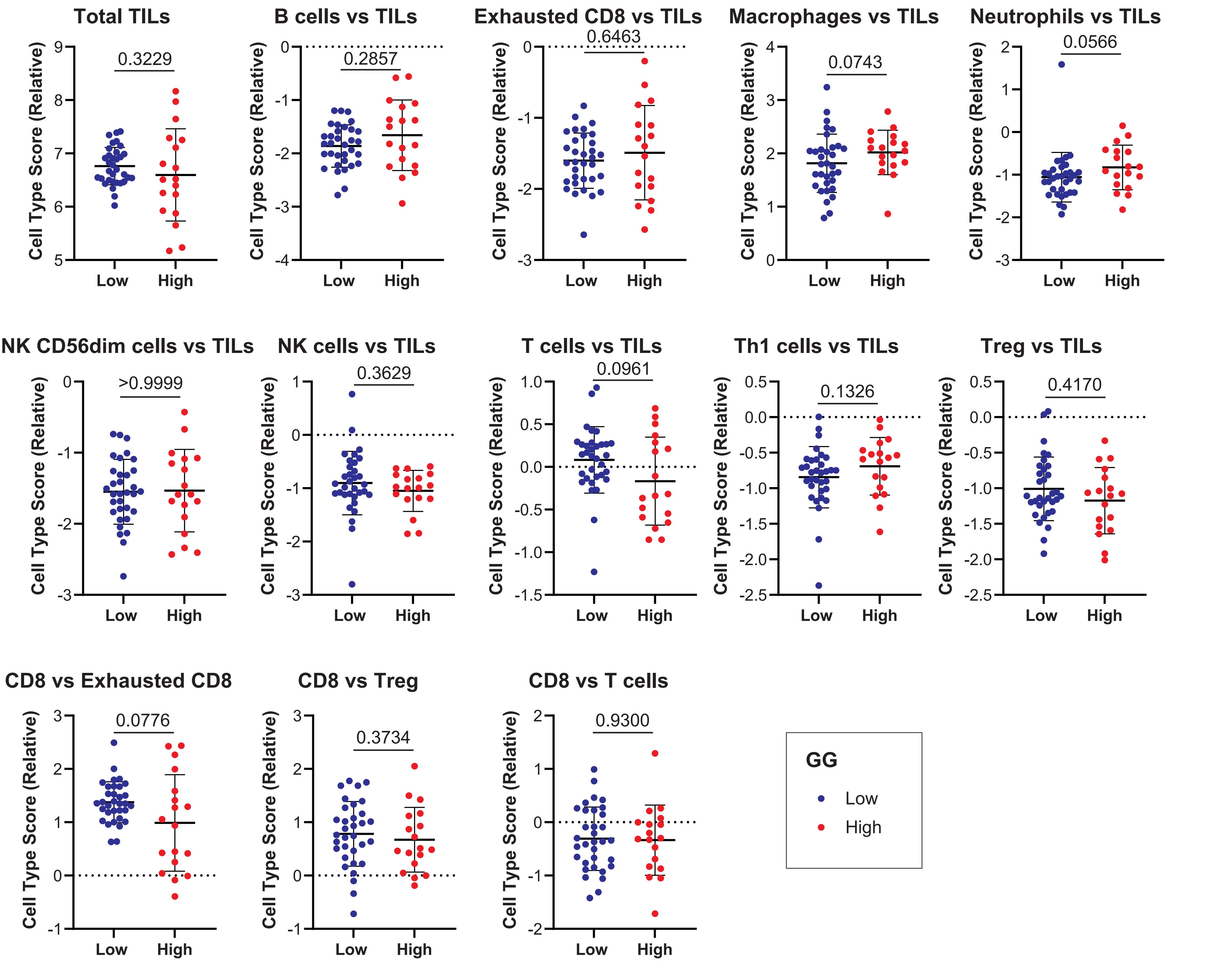

Supplementary Figure S4. Additional relative cell type contrasts compared by GG. Individual relative cell type scores are plotted for low GG (GG1-GG2) and high GG (GG4-GG5) tumors and are evaluated by Mann-Whitney U test. Each dot represents a patient score, and error bars correspond to mean ± SD.
