# Supplementary material for: Immunologic Assessment of Tumors from a Race-matched Military Cohort Identifies Mast Cell Depletion as a Marker of Prostate Cancer Progression
Source: Cancer Res Commun. 2023 Aug 1;3(8):1423–34. doi: 10.1158/2767-9764.CRC-22-0463 (PMC10392708; doi:10.1158/2767-9764.CRC-22-0463)
Supplement: Supplementary Figure S6 — shows non-significant relative cell type scores by Gleason Sum. [file crc-22-0463-s06.pdf]

Supplementary Figure S6

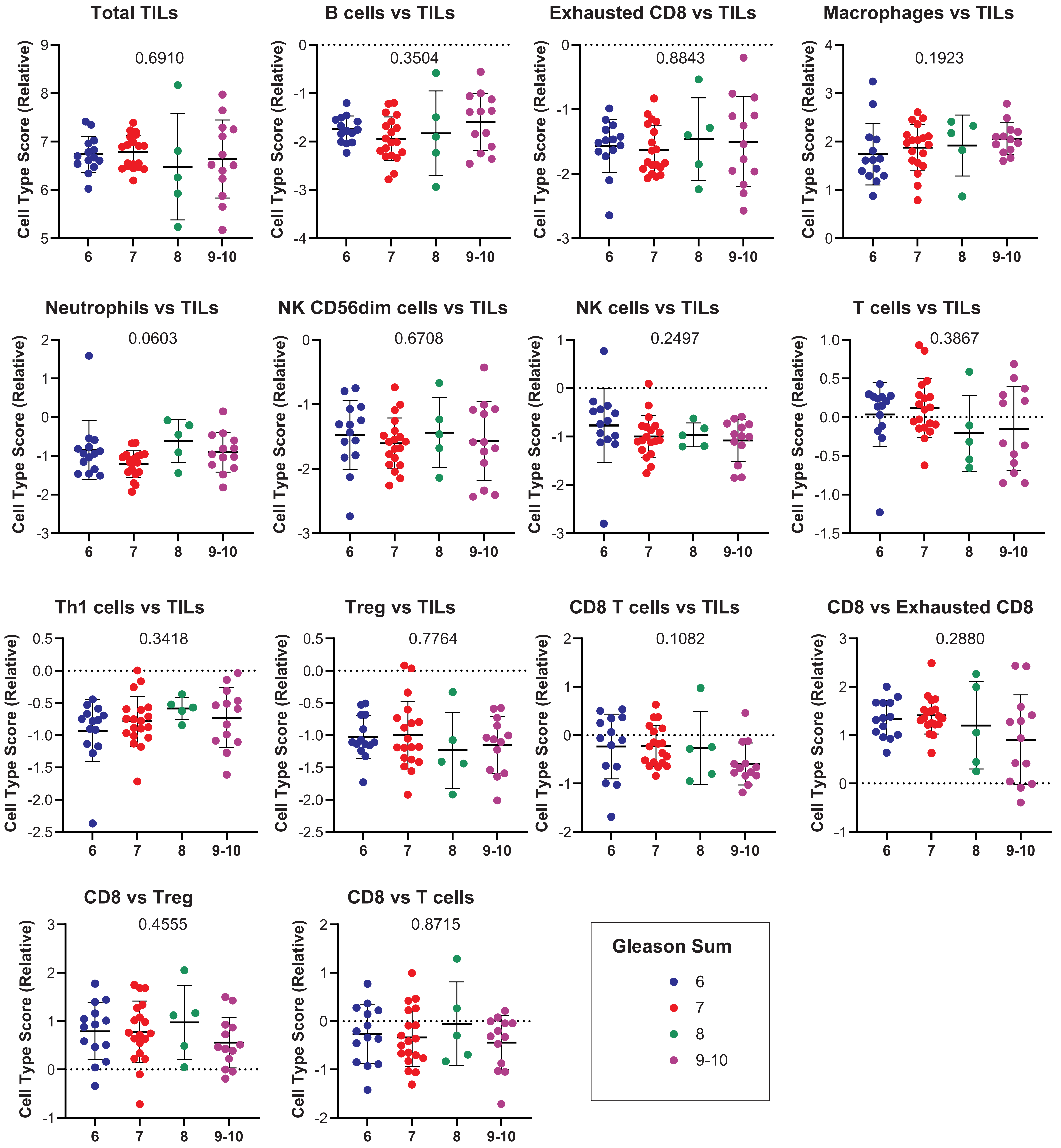

Supplementary Figure S6. Additional relative cell type contrasts compared by Gleason Sum. Individual relative cell type scores are plotted for Gleason Sum and are evaluated by Kruskal-Wallis test followed by Dunn's multiple comparisons post-test. Each dot represents a patient score, and error bars correspond to mean ± SD.
