# Supplementary material for: Immunologic Assessment of Tumors from a Race-matched Military Cohort Identifies Mast Cell Depletion as a Marker of Prostate Cancer Progression
Source: Cancer Res Commun. 2023 Aug 1;3(8):1423–34. doi: 10.1158/2767-9764.CRC-22-0463 (PMC10392708; doi:10.1158/2767-9764.CRC-22-0463)
Supplement: Supplementary Figure S7 — shows non-significant relative cell type scores by Dx PSA Group. [file crc-22-0463-s07.pdf]

Supplementary Figure S7

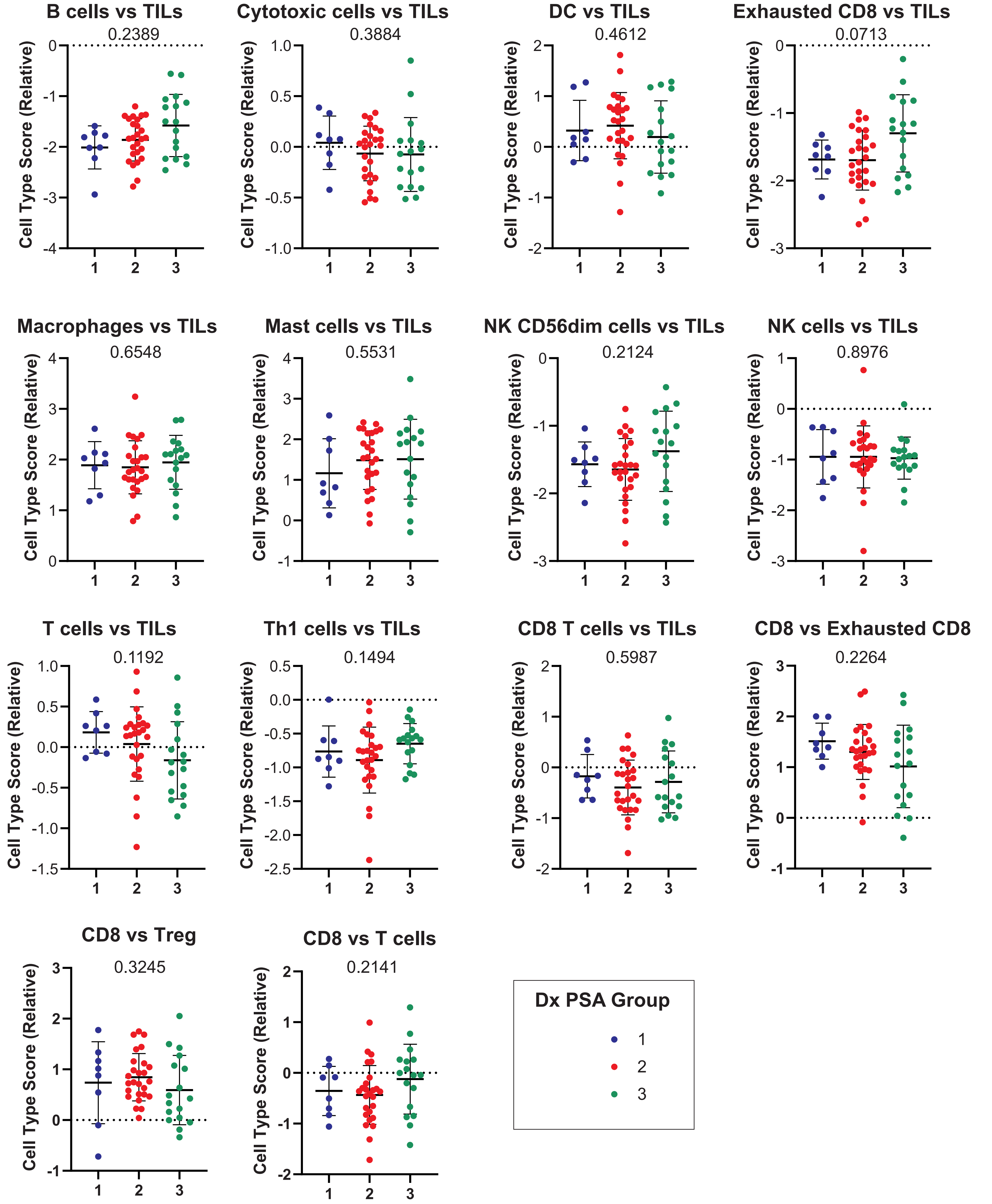

Supplementary Figure S7. Additional relative cell type contrasts compared by Dx PSA Group. Individual relative cell type scores are plotted for Dx PSA Groups and are evaluated by Kruskal-Wallis test followed by Dunn's multiple comparisons post-test. Each dot represents a patient score, and error bars correspond to mean ± SD.
