# Supplementary figures and images for: Immunologic Assessment of Tumors from a Race-matched Military Cohort Identifies Mast Cell Depletion as a Marker of Prostate Cancer Progression
Source: Cancer Res Commun. 2023 Aug 1;3(8):1423–34. doi: 10.1158/2767-9764.CRC-22-0463 (PMC10392708; doi:10.1158/2767-9764.CRC-22-0463)

# Supplementary Figure S8

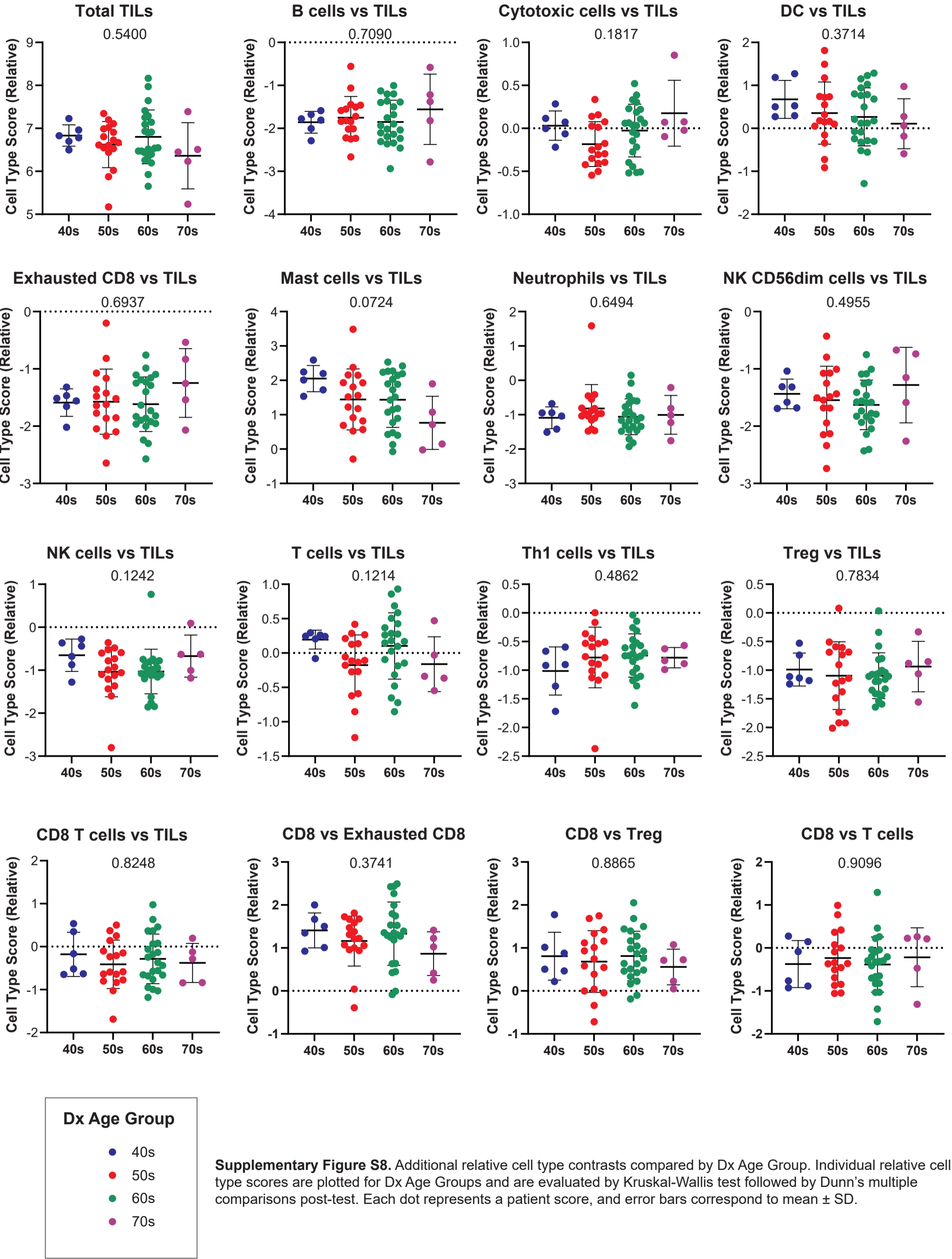

Supplement: Supplementary Figure S8 — shows non-significant relative cell type scores by Dx Age Group. [file crc-22-0463-s08.pdf]
