# Supplementary material for: Immunologic Assessment of Tumors from a Race-matched Military Cohort Identifies Mast Cell Depletion as a Marker of Prostate Cancer Progression
Source: Cancer Res Commun. 2023 Aug 1;3(8):1423–34. doi: 10.1158/2767-9764.CRC-22-0463 (PMC10392708; doi:10.1158/2767-9764.CRC-22-0463)
Supplement: Supplementary Figure S9 — shows BCR-free and Metastasis-free survival for Total TILs scores dichotomized by median as well as Youden index cutoffs. [file crc-22-0463-s09.pdf]

Supplementary Figure S9

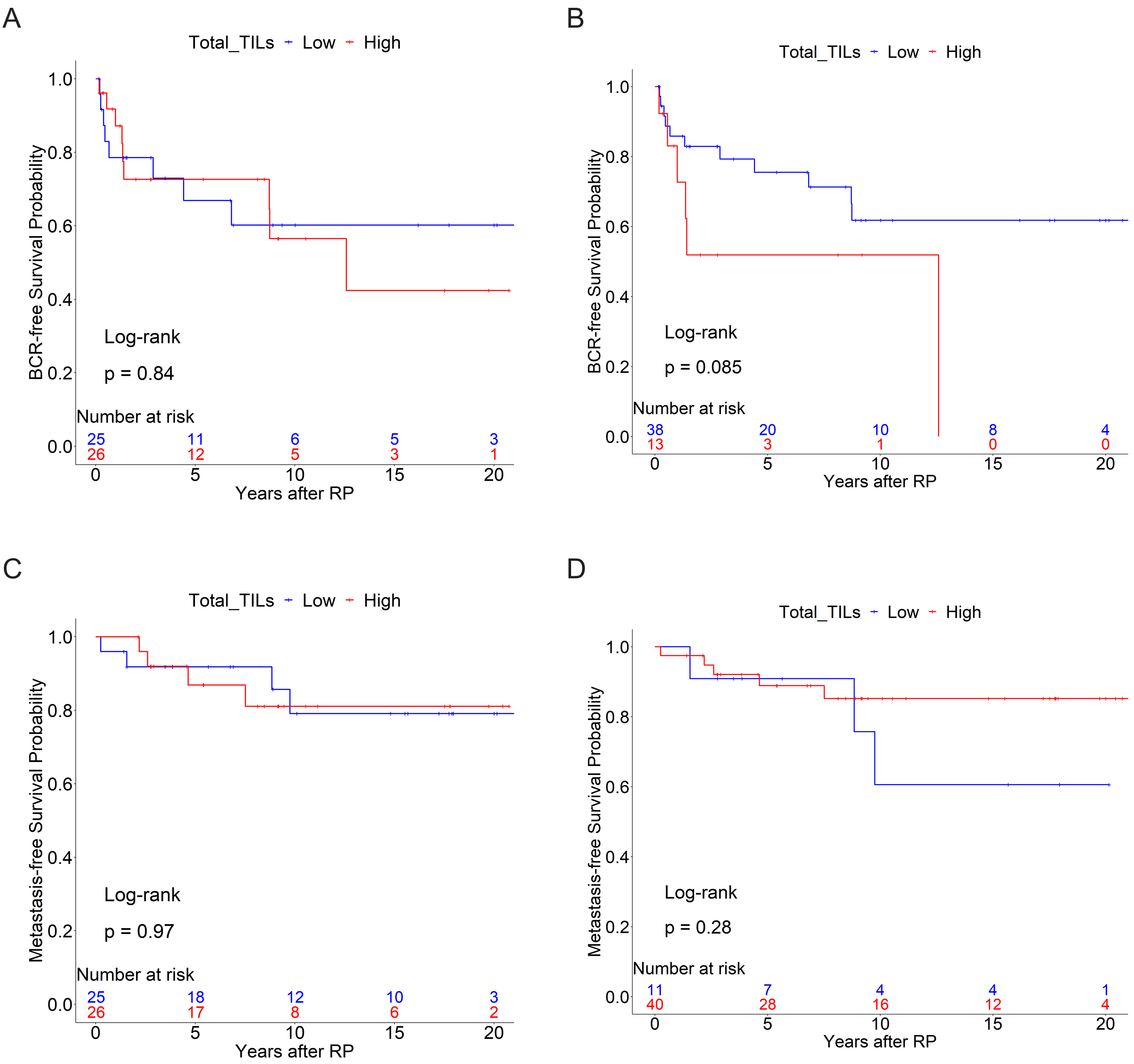

Supplementary Figure S9. BCR-free and metastasis-free survival differences in high vs low Total TILs scores. Kaplan Meier product-limit survival curves plotted for (A-B) BCR-free and (C-D) metastasis-free survival at log-rank p > 0.05 for high vs low Total TILs score cutoffs by (A and C) median and (B and D) Youden index.
