# Supplementary material for: Immunologic Assessment of Tumors from a Race-matched Military Cohort Identifies Mast Cell Depletion as a Marker of Prostate Cancer Progression
Source: Cancer Res Commun. 2023 Aug 1;3(8):1423–34. doi: 10.1158/2767-9764.CRC-22-0463 (PMC10392708; doi:10.1158/2767-9764.CRC-22-0463)
Supplement: Supplementary Figure S10 — shows BCR-free and Metastasis-free survival for top differentially expressed genes in AA tumors dichotomized by median cutoffs. [file crc-22-0463-s10.pdf]

Supplementary Figure S10

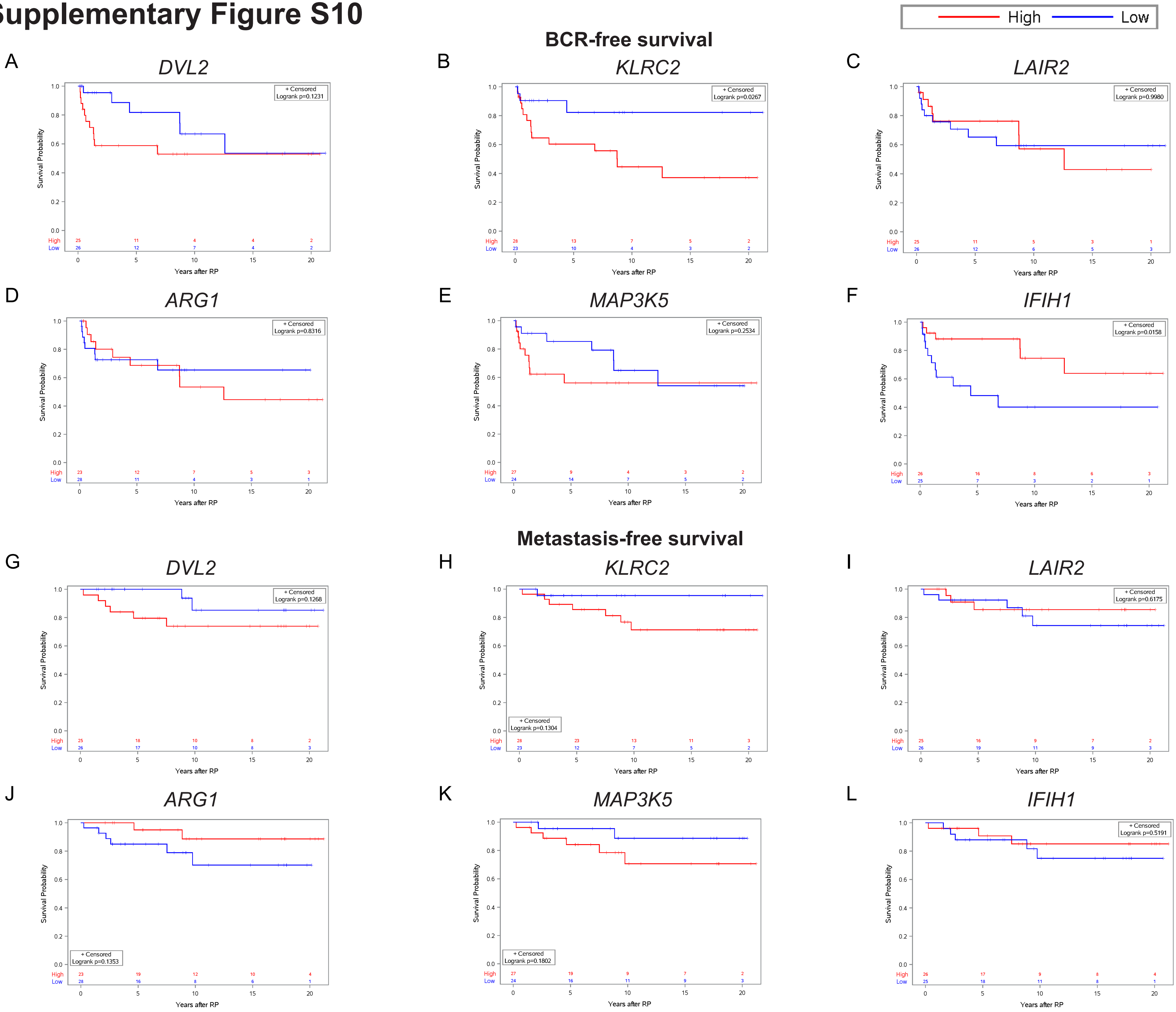

Supplementary Figure S10. Log-rank survival estimates, for (A-F) BCR-free and (G-L) metastasis-free survival, of the top six differentially expressed genes plotted by median cutoffs for high vs low gene expression. The full list of p-values for median, continuous, and Youden index analyses are shown in Supplementary Table S2.
