# Supplementary material for: Immunologic Assessment of Tumors from a Race-matched Military Cohort Identifies Mast Cell Depletion as a Marker of Prostate Cancer Progression
Source: Cancer Res Commun. 2023 Aug 1;3(8):1423–34. doi: 10.1158/2767-9764.CRC-22-0463 (PMC10392708; doi:10.1158/2767-9764.CRC-22-0463)
Supplement: Supplementary Figure S12 — shows Mast vs TILs scores plotted by race for Gleason Sum, GG, and Dx Age. [file crc-22-0463-s12.pdf]

Supplementary Figure S12

A

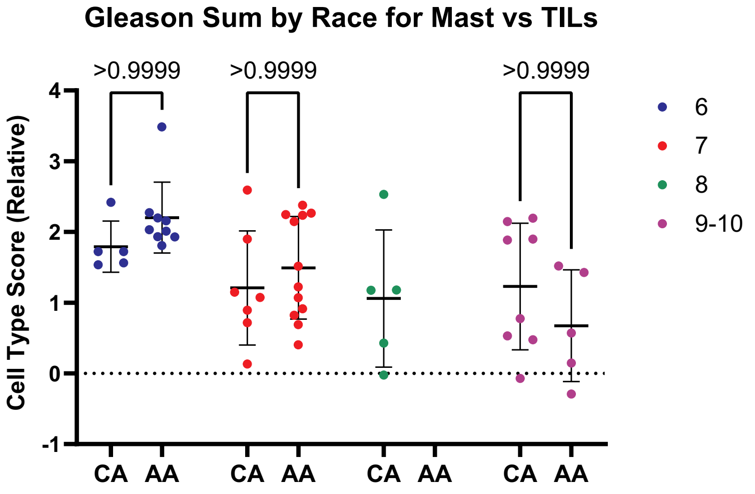

B

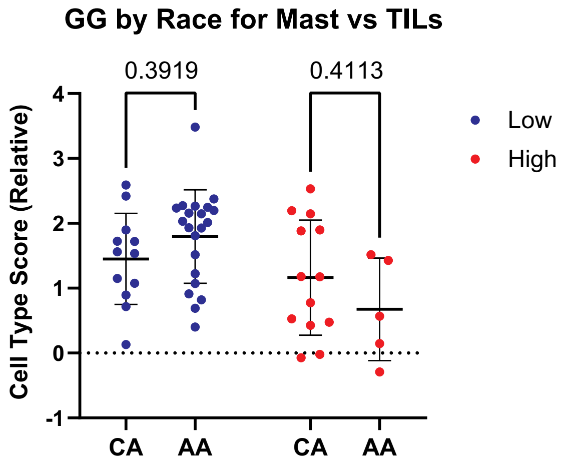

C

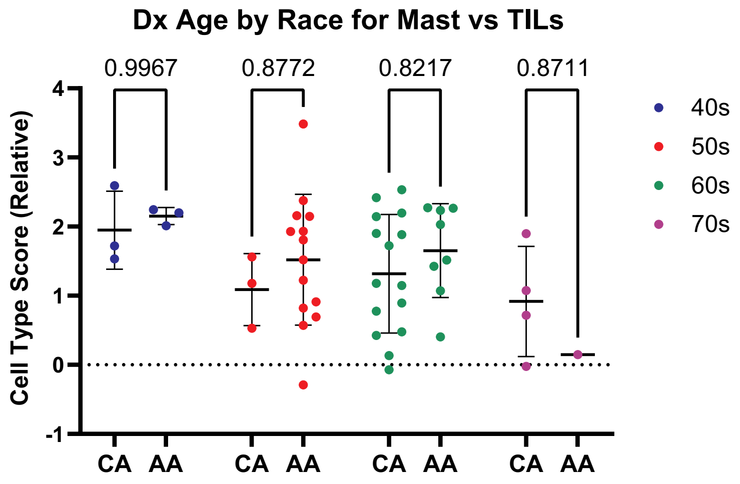

Supplementary Figure S12. Mast vs TILs scores stratified by race for (A) Gleason Sum, (B) GG, and (C) Dx Age. Error bars represent mean ± SD. 2-way ANOVA and Sidak's multiple comparisons test is used to compare groups with corresponding adjusted p-values.
